# Remote ischaemic conditioning for neurological disorders—a systematic review and narrative synthesis

**DOI:** 10.1186/s13643-024-02725-8

**Published:** 2024-12-19

**Authors:** Ali Alhashimi, Marharyta Kamarova, Sheharyar S. Baig, Krishnan Padmakumari Sivaraman Nair, Tao Wang, Jessica Redgrave, Arshad Majid, Ali N. Ali

**Affiliations:** 1 University of Sheffield, Sheffield, UK; 2 Department of Neuroscience, Geriatrics and Stroke, Sheffield Institute for Translational Neurosciences, University of Sheffield, Sheffield, UK; 3 Department of Neurology, Sheffield Teaching Hospitals NHS Foundation Trust, Sheffield, UK

**Keywords:** Remote ischemic conditioning, Ischemic conditioning, Stroke, Subarachnoid haemorrhage, Neurodegenerative disease, Vascular dementia

## Abstract

**Introduction:**

Remote ischaemic conditioning (RIC) refers to the use of controlled transient ischemic and reperfusion cycles, commonly of the upper or lower limb, to mitigate cellular damage from ischaemic injury. Preclinical studies demonstrate that RIC may have a neuroprotective effect and therefore could represent a novel therapeutic option in the management of neurological disorders. The aim of this review is to comprehensively describe the current clinical evidence of RIC in neurological disorders.

**Methods:**

A computerised search of EMBASE and OVID MEDLINE was conducted from 2002 to October 2023 for randomised controlled trials (RCTs) investigating RIC in neurological diseases.

**Results:**

A total of 46 different RCTs in 12 different neurological disorders (*n* = 7544) were included in the analysis. Conditions included acute ischaemic stroke, symptomatic intracranial stenosis and vascular cognitive impairment. The most commonly used RIC protocol parameters in the selected studies were as follows: cuff pressure at 200 mmHg (27 trials), 5-min cycle length (42 trials), 5 cycles of ischaemia and reperfusion (24 trials) and the application to the upper limb unilaterally (23 trials).

**Conclusions:**

The comprehensive analysis of the included studies reveals promising results regarding the safety and therapeutic effect of RIC as an option for managing neurological diseases. Particularly, the strongest evidence supports its potential use in chronic stroke patients and vascular cognitive impairment. The neuroprotective effects of RIC, as demonstrated in preclinical studies, suggest that this therapeutic approach could extend its benefits to various other diseases affecting the nervous system. However, to establish the efficacy of RIC across different neurological disorders, further trials with larger sample sizes and more diverse patient populations are warranted. Upcoming trials are expected to provide valuable evidence that will not only confirm the efficacy of RIC in neurological disease management but also help identify the most optimal RIC regimen for specific conditions.

**Supplementary Information:**

The online version contains supplementary material available at 10.1186/s13643-024-02725-8.

## Background

Cerebrovascular and neurological disorders account for a high proportion of global disability and mortality [1]. A significant proportion of this burden affects individuals from low- and middle-income countries. As such, the development of cost-effective treatments in stroke and other neurological diseases is essential to reducing inequality in healthcare outcomes.

Remote ischaemic conditioning (RIC) refers to a process whereby periods of temporary ischaemia, usually delivered by an occlusive blood pressure cuff set above systolic blood pressure to the upper or lower limb, confers a systemic protection against current or future ischaemic injury [2]. It has been shown to be a safe, well-tolerated procedure that has the potential to be a low-cost intervention in a number of vascular diseases [3–6]. Furthermore, there is a rapidly developing evidence base from animal models that indicate the benefits of RIC may extend to other neurological disorders such as spinal cord injury, traumatic brain injury and multiple sclerosis [7–9].

**Fig. 1:**
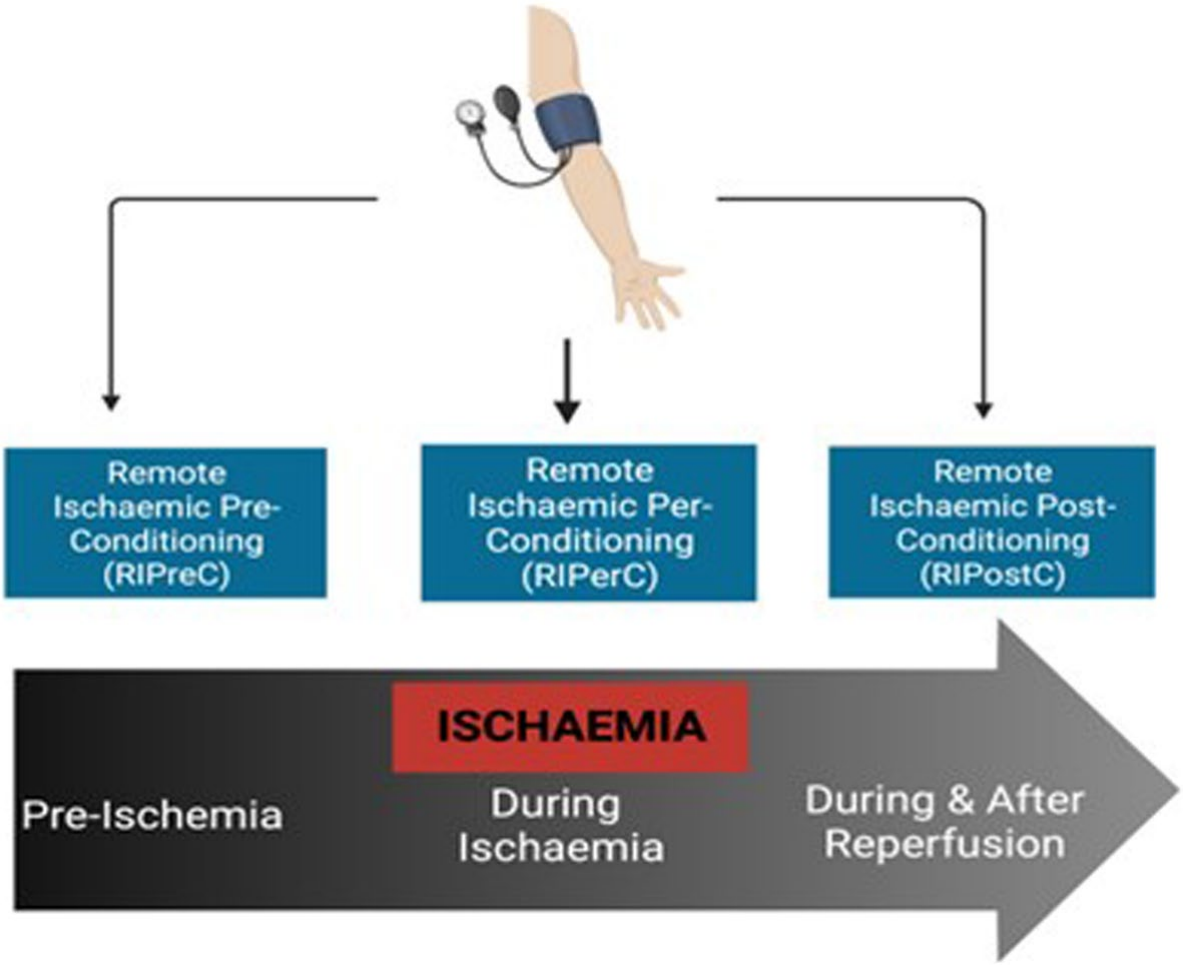
Paradigms of timing in remote ischaemic conditioning

RIC can be delivered prior to anticipated ischaemia (remote ischaemic preconditioning; RIPreC), during ischaemia (remote ischaemic perconditioning; RIPerC) or after ischaemia (remote ischaemic postconditioning; RIPostC). These paradigms are illustrated in Fig. 1. There are several putative mechanisms by which RIC may potentially improve clinical outcomes in stroke including increased angiogenesis [10], cerebral blood flow [11], reduced oxidative stress [12] and improved mitochondrial function [13]. These biological effects may widen the benefit of RIC to a range of neurological disorders where vascular insufficiency and impaired mitochondrial function are implicated in the pathophysiology [14, 15].

## Objectives

This systematic review examines the clinical evidence, from randomised controlled trials, of the benefits or harms of RIC as an additional therapy in participants with stroke, subarachnoid haemorrhage, vascular dementia and other neurological disorders.

## Methods

The systematic review followed the PRISMA reporting guidelines (Additional File 1).

### Search strategy

We searched MEDLINE via OVID and EMBASE via OVID from 2002 to 23rd October 2023. Search terms for “isch* conditioning,” “isch* preconditioning,” “isch* preconditioning” and “isch* postconditioning” were combined with the Cochrane highly sensitive search strategy for identifying randomised trials [16].

Only studies published from 2002 onwards were included; this time limit was selected as limb ischaemic conditioning was first described experimentally in clinically in neurological disease in 2002 [17]. No language restrictions were applied in the search strategy. Additional studies were identified through searching in reference lists, citation searches and conference proceedings. The full search strategy can be found in Additional File 2.

### Eligibility criteria and study selection

Titles and abstracts identified from the search strategy were independently screened by three reviewers (SB, MK and AALH). Duplicate articles were removed. Studies were eligible for inclusion in the systematic review according to the following predetermined criteria: they were a full-length article, written in English, a randomised controlled trial, the participants were individuals with cerebrovascular or neurological disease and the treatment included RIC. Where two reviewers disagreed on whether a study met the eligibility criteria, a fourth reviewer (AA) was consulted.

### Data collection process

Data extraction was performed independently by three authors (SB, MK and AALH) on an identical data collection template. Information was extracted on the methods, study participants, intervention, treatment duration, primary and secondary outcomes. The data collected was cross-checked between the authors and differences were resolved after referral to the source material.

### Assessment of bias

Three authors (SB, MK and AALH) independently assessed each study for the risk of bias using the Cochrane Collaboration’s tool for assessing risk of bias in randomised trials [18]. Risk of bias was assessed in six domains: selection bias (random sequence generation and allocation concealment), performance bias (blinding of participants and personnel), detection bias (blinding of outcomes assessors), attrition bias (incomplete outcome data), reporting bias (selective reporting) and other sources of bias. Studies were categorised as having low risk, unclear risk or high risk of bias. Where there was disagreement between the reviewers, a fourth reviewer (AA) was consulted.

### Synthesis and narrative review

Due to study heterogeneity in populations, RIC protocols and outcomes assessed, we did not perform a meta-analysis of outcome measures. We undertook a narrative synthesis of the available evidence using the framework published by the Cochrane Consumers and Communication Review Group [19].

## Results

### Study selection

We identified 7008 studies from the database search. After 2942 duplicates were removed, 4066 remained. We screened 4066 titles and/or abstracts for eligibility for inclusion in the review; 102 full-text articles were identified from the database searches and 4 additional full-text articles were identified from references and citation searches. One hundred and six full-text articles were assessed and 46 met the inclusion criteria for the narrative synthesis. The study selection flow diagram is illustrated in Additional File 2.

### Study characteristics

We identified 46 articles involving 7544 participants. The study characteristics are summarised in Table 1.

**Table 1:** Summary table of study characteristics

**Parameter**			**Number of Studies**
Study Population		Acute Ischaemic Stroke/TIA	19
		Symptomatic Intracranial Stenosis	5
		Ischaemic Small Vessel Disease	4
		Chronic stroke	3
		Carotid procedures	5
		Aneurysmal SAH	3
		Traumatic Brain Injury	1
		Cervical Spine Surgery	1
		Moya-Moya Disease	2
		Brain Tumour Surgery	1
		Intracerebral Haemorrhage	1
		Multiple Sclerosis	1
Timing of Conditioning		RIPreC	11
		RIPerC and early RIPostC	26
		Chronic RIPostC	9
Participants (N)		< 50	22
		51 to 100	14
		101–150	5
		151–200	3
		> 200	2
RIC Protocol	Cuff Pressure	180 mmHg	3
		200 mmHg	27
		225 mmHg	3
		25 mmHg > sBP	1
		20 mmHg > SBP	5
		30 mmHg > SBP	3
		110 mmHg > SBP	1
		Variable	2
		Until distal pulses occluded	1
	Cycle Length	3 min	2
		5 min	42
		10 min	1
		Variable	1
	No. cycles	2	1
		3	7
		4	14
		5	24
	Limb	Upper, unilateral	23
		Upper, bilateral	14
		Lower, unilateral	8
		Upper and lower unilateral	1
Study Location		Asia	28
		Europe	11
		North America	7

*RIPreC* Remote Ischaemic Pre-Conditioning, *RIPerC* Remote Ischaemic Per-Conditioning, *RIPostC* Remote Ischaemic Post-Conditioning

Conditions in which RIC was investigated were ischaemic stroke/TIA (19 trials), symptomatic intracranial stenosis (5 trials), ischaemic small vessel disease (4 trials), chronic stroke (3 trials), carotid stenosis (5 trials), subarachnoid haemorrhage (3 trials), Moyamoya disease (2 trials), traumatic brain injury (1 trial), brain tumour surgery (1 trial), cervical spine surgery (1 trial), intracerebral haemorrhage (1 trial) and multiple sclerosis (1 trial).

RIC protocols included bilateral upper limb conditioning (16 trials), unilateral upper limb conditioning (23 trials) and unilateral lower limb conditioning (7 trials) and a combination of upper and lower limb conditioning (1 trial).

In 28 trials, sham-RIC was the comparator, and in 17 trials, the comparator was usual medical/surgical therapy and 1 trial compared two different RIC protocols. The length of RIC therapy ranged between 1 and 360 days with the median length of therapy being 7 days. The follow-up ranged between no follow-up and 360 days, with the median follow-up being 90 days.

The outcome evaluation method varies depending on the specific disease being studied. Among the different methods used, clinical assessments are the most common, with a particular focus on the safety and feasibility of RIC as it was investigated in 23 trials. Two widely employed clinical assessment scales are the modified Rankin Scale (mRS) and the National Institutes of Health Stroke Scale (NIHSS), each utilised in 20 trials. These assessments provide valuable insights into the impact of treatments on patients’ neurological well-being and functional abilities. In addition to clinical assessments, radiological investigations are frequently employed to gain a deeper understanding of the mechanisms of action of RIC. Cerebral blood flow (cBF) measurement and imaging of ischaemic lesions or strokes have been utilised in 14 trials each. These radiological methods shed light on the physiological effects of RIC treatment, including its impact on blood flow, infarct volume and angiogenesis in the brain. Biochemical markers are also commonly used, with a particular focus on inflammatory markers like interleukin-6 (IL-6), interleukin-10 (IL-10), and C-reactive protein (CRP) and neuronal markers like s100B which were each measured in eight trials. The analysis of these markers provides crucial information about the underlying mechanisms of action of RIC. By understanding the biochemical pathways affected by the treatment, it may be possible to better tailor RIC usage and changes in these biomarkers could possibly be used to assess the therapeutic effects of RIC more accurately. Table 2 summarises the range of clinical, biochemical and radiological outcome measures assessed across studies.

**Table 2:** Outcome measures used in studies of RIC in neurological disorders

Clinical	Safety/feasibility	23
	Modified Rankin Scale	20
	NIHSS	18
	ADL	13
	Stroke/TIA/cardiac event	15
	Mortality	9
	Functional outcome/impairment	3
	Mood	4
	Muscle metrics	3
	Length of stay	2
	Walking metrics	3
	Seizure	1
	Quality of Life	1
Radiological	Cerebral blood flow	14
	Ischaemic lesions/stroke	14
	Infarct Size/Growth, penumbral salvage	7
	CNS haemorrhage	4
	White matter hyperintensities (WMH)	4
	Brachial artery flow-mediated dilatation	2
	Haematoma measurements	1
Cognition	Cognitive tests	7
Blood Biomarkers	Inflammatory	8
	Neuronal injury	8
	Miscellaneous	3
	Troponin	4
	Angiogenesis	2
	Coagulation	2
	Neurotrophic (BDNF)	2
Other	Heart rate variability	2
	P300 Event-related Potential	1
	Saccadic latency	1

ADL: Barthel, Karnofsky performance scale

Mood: Hamilton, Zung scales

Functional outcome/impairment: Fugl-Meyer, Dizziness Handicap Index, Japanese Orthopaedic Association Score

Walking metrics: speed, % improvement in 6 m walk test, Borg RPE

Muscle metrics: MRC, Muscular strength and fatigue (Biodex dynamometer), activity (EMG)

WMH: WML volume, Fazekas and Scheltens' scores, number of lacunes

Haematoma measurements: Haematoma volumes and resolution rates, perihaematomal oedema

Cerebral blood flow: Haemodynamics: pulsatility indices, MCA velocity and vasospasm on TCD; perfusion: SPECT; reperfusion: mTICI score; collat circulation: MRI, rLMC

Cognition: MMSE, MOCA, TICS-M, HVLT-R, COWAT, TMT-A & TMT-B and JLO

Neuronal injury: S100B, NSE

Inflammatory: MMP-9, CRP, ICAM-1, leukocytes, IL-6, TNFa, TLR4, NFKB, HSP27, homocysteine

Angiogenesis: VEGF, bFGF

Coagulation: D-dimer, TPA, PAI-1, fibrinogen, platelet aggregation

Misc: endocannabinoids, endothelin-1, triglycerides, cholesterol, haem-oxygenase 1

### Risk of bias

**Fig. 2:**
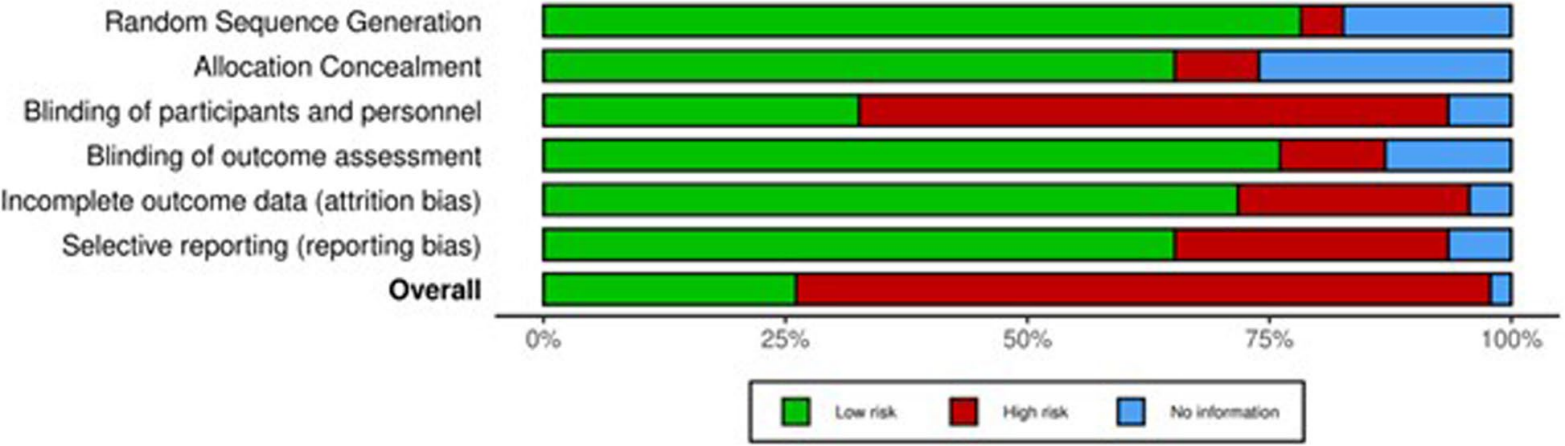
Cochrane risk of bias across studies of RIC in neurological disorders

The risk of bias within studies is presented in Additional File 3. The risk of bias across studies is presented in Fig. 2.

### Results of individual studies

The results of individual studies are summarised in an Excel data file available on request. A meta-analysis was not performed due to heterogeneity in study methods. Studies were clustered by the population of interest and synthesis of the findings is detailed in the “Discussion” below.

## Discussion

### Summary of evidence

#### Acute ischaemic stroke

The application of RIC in the setting of acute stroke is based on pre-clinical models which indicate that RIC can improve penumbral salvage and neurological outcomes [20, 21]. This has been postulated to occur through several mechanisms including increased cerebral blood flow [11], maintenance of dynamic cerebral autoregulation [22], promoting blood–brain barrier integrity [23] and reducing cerebral oedema [24].

Twelve RCTs have looked exclusively at the administration of RIC in the setting of acute ischaemic stroke [25–36]. The characteristics and main results of these studies are summarised in Table 3. It is important to note that there are significant methodological differences between the studies in their study populations, RIC protocols, timing of intervention, duration of intervention and outcome measures (clinical, biochemical and radiological). Given this variation, it is difficult to draw any definitive conclusions about the efficacy of RIC.

**Table 3:** Randomised controlled trials of Remote Ischaemic Conditioning in acute ischaemic stroke

**Study**	**N**	**RIC Protocol**					**Control Group**	**Main Results**
		**Limb**	**Pressure (mmHg)**	**Cycles**	**Duration**	**Timing**		
Hougaard et al. (2014) [25]	171	Unilateral non-paretic upper limb	200	4 × 5 min	Single treatment	Pre-hospital arrival in patients within 4.5 h of symptom onset	Standard medical therapy alone	No significant difference in penumbral salvage or infarct growth on MRI Reduced tissue risk of infarct on voxel based analysis
RECAST England et al. (2017) [29]	26	Unilateral, non-paretic upper limb	20 above SBP	4 × 5 min	Single treatment	Within 24 h of symptom onset	Sham RIC at 30 mmHg	Significant decrease in NIHSS score in RIC group at Day 90 compared to sham No difference in mRS at Day 90 RIC increased HSP-27 and phosphorylated HSP-27 at Day 4
Li et al. (2018) [23, 27]	68	Unilateral, non-paretic upper limb	20 above SBP	4 × 5 min	Daily up to 14 days/ discharge	24–72 h post symptom onset	Sham RIC at 30 mmHg	Lower NIHSS and mRS at Day 90 compared to sham in RIC group Lower infarct volume and higher regional relative cerebral blood flow on MRI at Day 90 in RIC group
Che et al. (2019) [30]	30	Bilateral upper limb	200	5 × 5 min	Twice daily up to 6 days	Within 2 h post- thrombolysis	Standard medical therapy alone	No difference in NIHSS, Barthel Index or mRS at Day 90
RECAST2 england et al. (2019) [31]	60	Unilateral, non-paretic upper limb	20 above SBP	4 × 5 min	Once, twice or twice daily 4 days	Within 6 h of symptom onset	Sham RIC at 20 mmHg	No significant difference in stroke extension on stroke recurrence at Day 90 Increased levels of S100β from Day 1 to Day 4 in sham group but not in RIC group
An et al. (2020) [28]	68	Bilateral upper limb	180	5 × 5 min	Twice daily throughout admission	Within 3 h post-thrombolysis	Standard medical therapy alone	Higher proportion of mRS 0–1 in RIC group at day 90 Higher proportion with a 6 point improvement in NIHSS at day 90.improvement in NIHSS at day 90 Lower S100β and higher VEGF in RIC group at discharge
RESCUE-BRAIN Pico et al. (2020) [26]	188	Unilateral non-paretic lower limb	110 above SBP	4 × 5 min	Single treatment	Within 6 h of symptom onset	Standard medical therapy alone	No significant difference in MRI infarct volume or NIHSS at 24 h No significant difference in mRS or Barthel Index at Day 90
RICAMIS Chen et al. (2022) [35]	1776	Bilateral upper limb	200	5 × 5 min	twice a day for 10–14 days	enrolled within 48 h of stroke symptom onset	Standard medical therapy alone	significant increase in proportion of patients with mRS of 0–1 in RIC group (adjusted for confounding) significant increase in the proportion of patients with mRS of 0–2 in RIC group
He et al. (2022) [34]	126	Unilateral upper limb	200	3 × 5 min	once a day for 7 days	duration from symptom onset to treatment < 3 h	tPA + Standard medical therapy alone	The NIHSS and mRS score were significantly lower than those in the control group and before treatment GOS score in the experimental group was significantly higher no significant difference in the incidence of adverse reactions
REPOST Landman et al. (2023) [33]	88	Unilateral upper limb	200	4 × 5 min	twice a day for 4 days	enrolled within 24 h of stroke symptom onset	Sham RIC at 50 mmHg	No significant difference in the median infarct volume after 4 days No significant change in mRS score and NIHSS score after 12 weeks
Poalelungi et al. (2021) [32]	40	unilateral upper limb	20 above sbp	5 × 3 min	twice daily for 5 days	Within 24 h of symptom onset	Sham RIC at 30 mmHg	No significant difference in infarct volume after 6 months no significant difference in clinical scores (NIHSS, mRS, ADL) and cognitive/mood changes (MoCA, PHQ-9) at 180 days compared to baseline
Wang et al. (2022) [36]	86	Bilateral upper limb	180	5 × 5 min	once a day for 7 days	enrolled within 48 h of stroke symptom onset	Sham RIC at 60 mmHg	Significant decrease in NIHSS score and and modified Rankin scale significantly increased after 7 days

*HSP-27* Heat Shock Protein 27, *mRS* Modified Rankin Scale, *S100β* S100 Calcium-binding protein beta, *SBP* Systolic Blood Pressure, *VEGF* Vascular Endothelial Growth Factor

Hougaard et al. first demonstrated the safety and feasibility of delivering in stroke patients with a proof-of-concept open label study of pre-hospital, unilateral, non-paretic arm RIC in individuals suspected of having acute stroke [25]. Whilst there was no significant effect of a 4 × 5 min cycles of RIC on the MRI endpoints of penumbral salvage, infarct growth or final infarct size, in a voxel-based logistic regression analysis, RIC was associated with reduced tissue risk of infarction [25]. RESCUE BRAIN, a relatively large multi-centre study of RIC in acute ischaemic stroke, demonstrated that there was no significant difference in the percentage change in MRI infarct volume at 24 h with RIC compared to standard medical therapy [26]. Furthermore, RIC was not associated with any improvement in NIHSS at 24 h or mRS at 90 days. This is in contrast with smaller studies from Li et al. (2018) (*n* = 68) and An et al. (2020) (*n* = 68) which demonstrated that RIC was associated with improvements in mRS at Day 90 [27, 28]. Importantly, in these latter studies, RIC was repeated daily for up to 14 days rather than a single treatment. It is therefore possible that repeated cycles of RIC may be of greater benefit than a single treatment in the setting of acute ischaemic stroke.

The studies of RIC in acute ischaemic stroke have investigated several blood-borne factors which may explain the mechanism of RIC and/or be candidate biomarkers for monitoring the effect of RIC on an individual level. The RECAST trial demonstrated increased levels of Heat Shock Protein 27 (HSP-27) following RIC [29]. Heat shock protein 27 has been shown to protect against ischaemic injury in pre-clinical models of cardiovascular disease [37]. There is evidence that RIC either reduces or attenuates the rise of S100β in ischaemic stroke [28, 29]. S100β usually rises following ischaemic stroke; this may be proportionate to the magnitude of infarction and associated with blood–brain barrier permeability [38, 39]. As such, reductions in S100β following RIC may signify improvements in blood–brain barrier integrity and reduced neuronal damage. Finally, the rises in vascular endothelial growth factor (VEGF) suggest that RIC may potentially increase angiogenesis in the post-stroke period which, in turn, may impact on post-stroke recovery [28, 29].

The RICAMIS trial is the largest randomised control trial of RIC in ischaemic stroke with 1776 patients recruited. It utilised an intensive protocol of 5 × 5 min cycles of bilateral upper limb RIC at 200 mmHg, twice a day for 2 weeks [35]. The results showed significant improvement in neurological function demonstrated by a higher proportion of individuals with an mRS score of 0–1 at 90 days when treated with RIC compared to patients receiving standard therapy (582 (67.4%) in the RIC group and 566 (62.0%) in the control group; odds ratio, 1.27 [95% CI, 1.05–1.54]; *P* = 0.02). This result supports the hypothesis that repeated cycles have a more prominent neuroprotective effect as seen in earlier trials [27]. The secondary outcome of the RICAMIS trial showed no significant improvement in neurological function in the short term; the study posited that RIC had a more neurorestorative effect over a long period of time rather than a more immediate neuroprotective effect on penumbral tissue [27]. This was supported by multiple RCTs showing no significant immediate improvement in neurological function and radiological measures of ischaemic stroke [26, 27, 33, 40]. However, a recent trial conducted by Wang et al. provided contradictory evidence; the results show a statistically significant improvement in NIHSS and mRS score at 8 days after starting RIC when compared to control and baseline data [36]. Trial participants underwent 5 cycles of 5 min of bilateral upper limb ischaemic conditioning at 180 mmHg once a day for 7 days. The protocol used was relatively aligned with previous studies, with the exception of a less frequently used reperfusion time of 3 min. The influence of this variation cannot be inferred from current data. Larger trials are needed to determine the impact of each parameter and therefore the most optimal protocol for RIC in the management of acute ischaemic stroke.

Post hoc analyses were conducted on RICAMIS data [41–43]. The first analysis examined the effect of patient sex on RIC efficacy. For the primary outcome (mRS scores of 0–1 within 90 days), RIC yielded an adjusted OR of 1.379 [95% CI 1.045–1.820] in men and 1.628 [95% CI 1.134–2.339] in women [41]. Secondary outcomes showed no sex differences. Whilst RIC improved functional outcomes in both sexes, females saw a notably greater improvement in primary outcomes. The second analysis looked at the relationship between onset to treatment time (OTT) and RIC outcomes [42]. Of the 863 patients in the RIC group, 387 received RIC within 24 h of symptom onset (early RIC group) and 476 received RIC after 24 h. The early RIC group had 70.0% of its patients achieve mRS scores of 0–1 at 90 days, whilst the late RIC group achieved 65.3% and the control group 62.0%. The early RIC group significantly outperformed the control, whereas the late RIC group did not. The secondary outcome of proportion of patients having mRS scores of 0–2 mirrored the primary results, but there were no significant differences in other secondary outcomes among the three groups. These findings align with prior studies emphasising the benefits of early RIC introduction [24, 28, 29]. The third analysis looked at the impact of diabetes and raised fasting blood glucose (FBG) on RIC’s therapeutic effect [43]. A larger proportion of non-diabetic patients achieved better functional outcomes (mRS 0–1) with RIC than diabetic patients (7.3% vs. 5.5%). Similar trends were observed concerning FBG. Earlier trials have indicated that diabetes and hyperglycemia diminish RIC’s cardioprotective effects [44]. This study revealed a similar impact on RIC's neuroprotective effect.

Three trials have explored the efficacy of RIC as adjunct therapy to thrombolysis in the management of acute ischaemic stroke [28, 30, 34]. Animal models have shown that a combination of IV thrombolysis and RIC can significantly reduce infarct volume and improve neurological outcome [45, 46]. The first clinical trial conducted by Che et al. (2018) showed that combination therapy significantly improved neurological outcomes at 30 days when compared to tPA only [30]. However, no significant difference was seen between both groups at 90 days, this is due to the small nature of the study and all participants scoring less than 15 on NIHSS at baseline and therefore have good prognosis over 3 months, thus making it difficult to ascertain the efficacy of RIC as most patients show significant or full recovery with thrombolysis alone. The results showed that combination therapy accelerated recovery as a significant improvement can be seen at 30 days when compared to control but similar results at 90 days. Larger trials with more severe NIHSS-scoring patients are needed to assess the impact of RIC in combination with thrombolysis. An et al. (2020) conducted the second trial exploring the effectiveness of tPA with RIC, this trial recruited 68 patients scoring less than 25 on NIHSS [28]. The results show marked improvement in the mRS score at 90 days and also showed significantly lower s100b and higher VEGF when compared to baseline and control at discharge. Biomarker results suggest that the combination of RIC and tPA confers a neuroprotective effect on the central nervous system. The final trial conducted by He et al. (2022) showed that RIC administered 2 to 3 h after thrombolysis causes significant improvement in NIHSS and Glasgow Outcome Score (GOS) at follow-up 6 months after treatment, with no significant increase in adverse events [34]. Whilst the exact mechanism of RIC is still being explored, preclinical studies have suggested that RIC complements thrombolysis treatment as it confers local central nervous system (CNS) resistance to reperfusion injury by prolonging local activation of the Akt pathway [45–47]. Activation of the Akt pathway has been shown to significantly reduce infarct volume by upregulating nitric oxide, therefore promoting endovascular homeostasis [46, 47].

Whilst animal studies provide sufficient evidence to the efficacy of RIC in the management of ischaemic stroke, more robust clinical trials are needed to identify optimal RIC parameters in the management of acute ischaemic stroke in patients. Current data suggest RIC is a safe and effective measure that can be used in conjunction with established treatments to significantly improve neurological outcomes and prognosis. Further qualitative studies on the experience of applying RIC in time-critical situations such as delivery of thrombolysis and mechanical thrombectomy will be an important guide to the practical aspects of therapy delivery in clinical practice.

### Subacute and chronic stroke

There are two distinct paradigms for the application of RIC to subacute and chronic stroke. The first is centred around using repeated cycles of RIC to induce protective effects against future vascular events. The second concerns the potential for RIC to improve functional recovery in both subacute and chronic stroke.

#### Stroke recurrence

The majority of studies investigating the utility of RIC in the prevention of recurrent stroke involve repeated, daily cycles of RIC in populations with symptomatic intracranial stenosis [48–52]. This was first demonstrated by Meng et al. who randomised 103 individuals with symptomatic intracranial stenosis to either twice daily, bilateral upper limb RIC for 300 days or usual medical therapy. The proportion of individuals who developed recurrent stroke at 300 days was 7.9% in the RIC group compared to 26.7% in the control group. Furthermore, there were no major, treatment-related adverse events. Similarly, daily RIC for 180 days in individuals aged 80–95 with symptomatic intracranial stenosis was associated with a lower frequency of recurrent stroke and TIA compared to sham RIC [48].

Pre-clinical studies have identified several mechanisms by which chronic RIC may mitigate the impact of vascular events [53]. These include improved mitochondrial function [13, 14], reduced platelet activity [54, 55], increased angiogenesis and cerebral blood flow [11, 45], improved endothelial function and reduced oxidative stress and systemic inflammation [56–58]. Some of these mechanisms have been substantiated in clinical studies of RIC. With regard to cerebral blood flow, RIC has been associated with increased cerebral perfusion on SPECT, reductions in peak systolic blood flow velocity at sites of stenosis on transcranial Doppler [48] and increases in mean blood flow velocity in the major intracranial vessels [51]. Increases in VEGF seen 10 days after RIC indicate that the improvements in cerebral blood flow may be driven by increased collateral circulation [50]. Meng et al. demonstrated that RIC was associated with an increase in plasma levels of tissue plasminogen activator and reductions in fibrinogen and plasminogen activator inhibitor-1 (PAI-1) indicating that repeated cycles of RIC may alter the balance between thrombosis and haemostasis towards a reduction in ischaemia [49]. Whilst there is some evidence that RIC may improve brachial artery flow mediated dilatation (FMD), a marker of endothelial function, in individuals with chronic stroke [59], the ERICS trial did not demonstrate any improvements in FMD after 12 weeks of RIC in patients with recent ischaemic stroke [40].

Meng et al. also demonstrated that RIC may accelerate neurological recovery from stroke [49]. After 180 days of bilateral upper limb RIC in individuals aged 80–95 with symptomatic intracranial stenosis, the average NIHSS and mRS scores in the RIC-treated group were 2.97 ± 1.97 and 1.4 ± 1.0, respectively, which were both significantly lower than the control group values of 4.82 ± 2.72 and 2.3 ± 1.1 (all *p* < 0.01). Consistent with this, Xu et al. found that 6 months of RIC in symptomatic intracranial stenosis, in addition to reducing risk of new infarcts, was also associated with a higher likelihood of improvement in function compared to sham (as measured by the New Injury Severity Score and Barthel Index) [51]. In this study, RIC was associated with increased cerebral blood flow velocity, reduced MMP-9 and increased BDNF suggesting that improved cerebral perfusion, blood–brain barrier integrity and neurogenesis may underlie the effect of long-term RIC in neurological recovery after stroke. It is important, however, to note that the improvement in neurological function may relate to spontaneous recovery after stroke and the reduction in risk of further infarction rather than necessarily being due to direct effects on neurological recovery.

The RICA trial conducted by Hou et al. is the largest RIC trial in neurology. It randomised 3033 patients with symptomatic intracranial atherosclerotic stenosis [52]. The intervention group received 5 cycles of 5-min bilateral upper limb RIC at 200 mmHg once daily for 12 months and the control group received sham RIC at 60 mmHg. The results show no significant difference in the incidence of stroke during the first 12 months between both groups; however, the study did report an issue with compliance due to the inconvenience of daily RIC; only 1409 (46.5%) patients were compliant with 50% or more of the intervention during the first 12 months. When comparing patients who were compliant with 50% or more of the intervention, a significant reduction in the incidence of stroke can be seen. The trial also showed a significant reduction in the cumulative incidence of stroke, TIA and myocardial infarction in the intervention group. These findings further substantiate the Importance of consistent RIC therapy in potentially eliciting a neuroprotective effect. The development of biomarkers of RIC activity and neuroprotection may enable participants to use RIC at more manageable doses and thereby encourage concordance.

#### Stroke recovery

RIC has also been utilised in chronic stroke survivors with persistent and stable neurological deficits as a potential tool to promote functional recovery [59–61]. In one study, 2 weeks of RIC in chronic stroke survivors with residual lower limb weakness was shown to increase self-selected walking speed in a 10-m walk test and increase the time to fatigability at 30% maximum voluntary contraction of the knee extensors in the paretic leg [59]. It is possible that this relates to improvements in skeletal muscle blood flow or the autonomic response to exercise as has been demonstrated in healthy volunteers [62]. The same protocol was found to increase brachial artery flow-mediated dilatation suggesting that RIC may improve endothelial function in chronic stroke survivors [60]. It has not been demonstrated whether repeated cycles of RIC in chronic stroke survivors lead to any cortical changes that aid in neurological recovery from stroke.

RIC also has the potential to mitigate common complications associated with stroke. Post-stroke cognitive impairment (PSCI) has been shown to affect 20–80% of stroke patients, a large variation in population prevalence is due to different diagnostic criteria in different regions [63]. Previous trials have shown that RIC can enhance post-surgical cognitive function, with speculation that its beneficial effects are due to its inhibitory impact on inflammatory processes [64, 65]. Feng et al. studied the impact of RIC administered in the acute phase of stroke management on the development of PSCI [66]. Results showed significant reduction in the incidence of PSCI at 6 months, with 54% of patients in the control group developing PSCI compared to 28% in the RIC group. Significant improvements were also observed in MoCA and ADL scores at 6 months. Interestingly, marked improvements were seen in the domain scores of ‘visuospatial and executive functioning’ and ‘attention’ [66]. Additionally, p300 latency and magnitude measurements were used to assess cognitive function; it is a reliable and sensitive test in identifying mild cognitive impairment [67]. The results show significantly shorter latency and larger magnitudes in the RIC group. The study also showed significant improvement in cerebral blood flow, supporting previous studies that show RIC enhancing cerebral blood flow [11, 45, 48–51]. Additionally, a reduction in biochemical markers intracellular adhesion molecule-1 (ICAM-1) and endothelin-1 (ET-1) were seen in the intervention group, both indicators of inflammation and endothelial dysfunction, further supporting the proposed mechanism of action of ischaemic conditioning [56–58]. The therapeutic effect of RIC on PSCI is corroborated by Li et al., and they showed significant improvement in MoCA and ADS-cog at 180 days post stroke [68]; 75% of patients in the RIC group were responders (ADAS-cog improvement of ≥ 4 points) compared to 45.8% in the control group.

Another common post-stroke complication is stroke-associated pneumonia (SAP), which has a prevalence of 14.3% and has been shown to be the third most common post-stroke complication [69, 70]. It stands as the one of the most common causes of death during the acute period of ischaemic stroke, with a threefold increase in mortality rate in the first 30 days [71]. Zhang et al. demonstrated that RIC during the acute phase of ischemic stroke has the potential to reduce the incidence of SAP [72]. The trial exhibited a decrease in incidence from 27.5% in the control group to 10.3% in the RIC group; however, due to the small scale of the RCT, this data was not statistically significant [72]. Stroke-associated immunosuppression predisposes patients to SAP; therefore, the levels of human leukocyte antigen-DR isotype (HLA-DR), toll-like receptors (TLR) 2 and 4 were also measured [73]. A reduction in HLA-DR has been strongly associated with SAP and immunosuppression [74], and although HLA-DR levels increased at 5 days, they were not statistically significant when compared to the control group. TLR 2 and 4 are key receptors in the inflammatory cascade and have previously been linked to the inflammatory process in stroke [75, 76]. Whilst TLR levels were not significantly impacted by RIC, the trial did show a significant reduction in proinflammatory cytokines interleukin 1-beta (IL-1B) and interleukin -6 (IL-6), supporting previous clinical trials and animal studies that demonstrate RIC’s ability to modulate inflammatory processes [75–77].

In a recent pilot trial conducted by Moyle et al. (2023), the safety and therapeutic impact of RIC on post-stroke fatigue (PSF) was investigated [78]. PSF, defined as a multidimensional experience encompassing motor-perceptive, emotional and cognitive aspects, often resulting in early exhaustion, weariness and aversion to effort [79], affects approximately 50% of stroke survivors and has been linked to higher mortality rates and reduced quality of life [80, 81]. The study’s outcomes align with prior trials, demonstrating the safety of RIC in stroke patients, with no serious adverse effects noted in either group. Mild, transient adverse effects were observed in up to 45% of patients in the RIC arm but it did not discourage participants from continuing the trial. The study employed the 7-item Fatigue Severity Scale (FFS-7) to assess fatigue symptoms, revealing a reduction in FFS-7 scores among RIC recipients compared to controls at 6 weeks, a reduction that persisted at 3 and 6 months; however, this was not statistically significant. Additionally, the trial utilised the 6-min walk test (6MWT) to quantify fatigue; a significant improvement of 48.3 m was seen when comparing the RIC group to the control group. Cardiopulmonary exercise testing (CPET) and phosphorous-31 magnetic resonance spectroscopy (P-MRS) were employed to explore the underlying mechanisms of RIC and its effect on PSF. CPET results indicated a reduction in the minute ventilation/carbon dioxide slope (VE/VCO2), suggesting enhanced capacity for oxidative metabolism and anaerobic glycolysis. P-MRS imaging revealed improved mitochondrial function, with all RIC participants displaying increased adenosine triphosphate (ATP) levels in the stroke-affected limb compared to a reduction in the sham group. In conclusion, whilst this pilot study may have been underpowered, it provides promising insights into the potential of RIC in alleviating fatigue in stroke patients and explores the underlying biological mechanisms driving its therapeutic effects on PSF.

#### Cerebral small vessel disease and vascular cognitive impairment

Cerebral small vessel disease (cSVD) is a heterogeneous pathological process, affecting arterioles, capillaries and venules that supply deep white matter in the brain. It is characterised radiologically as white matter hyperintensities, lacunar strokes and microbleeds, and driven by typical vascular risk factors (hypertension, high cholesterol, diabetes, smoking and poor cerebral perfusion). Although the radiological appearances can be asymptomatic in some individuals, cSVD contributes to 25% of ischaemic strokes and is correlated with cognitive decline [82].

Four studies have investigated the effect of twice daily RIC versus a sham procedure on small vessel disease over a period of 6 to 12 months [83–86] and are summarised in Table 4. All studies were based in China and had differing patient populations, including patients with non-genetic cSVD [83], mild cognitive impairment caused by cSVD [84], vascular dementia according to the Diagnostic and Statistical Manual of Mental Disorders 4th Edition (DSM-4) [85] and intracranial atherosclerotic stenosis in octo- and nonagenarians [86]. All studies have shown results favouring RIC on measures of either neuroimaging, cognitive testing, cerebral haemodynamics or blood biomarkers.

**Table 4:** Comparison of studies of RIC in cSVD or vascular cognitive impairment

**Author**	**Population**	**RIC Protocol**	**Baseline cognition of RIC group**	**WMH**	**Cognition**	**Pulsatility Index**	**Blood biomarkers**
Mi	Non-genetic cSVD	Upper limb (BL) 5 cycles, 200 mmHg, twice daily for 1 year	MMSE 30^a^ MoCA 26^a^	↓	↔	↔	
Wang	MCI in cSVD	Upper limb (BL) 5 cycles, 200 mmHg, twice daily for 1 year	MMSE 28.6 MoCA 24.9	↓	↔ (↑)^b^	↓	↓
Liao	Vascular dementia	Upper limb (BL) 5 cycles, 200 mmHg, twice daily for 6 months	MMSE 23.4 MoCA 18.3	↔	↑^c^		↔
Zhou	AIS	Upper limb (BL) 5 cycles, 200 mmHg, twice daily for 300 days	MMSE 24.3 MoCA 22.2	↓	↑		

*AIS* acute ischaemic stroke, *cSVD* cerebral small vessel disease, *MCI* Mild Cognitive Impairment, *B/L* bilateral

^a^Median

^b^Improvement in visuo-spatial and executive function of MoCA only

^c^Improvement in Verbal Learning Test (VLT-R), Controlled Oral Word Association Test (COWAT), Trail Making Tests A & B (TMT-A & B) and Judgment of Line Orientation (JLO)

Baseline cognition scores were comparable for RIC vs sham in all studies

Mi et al. and Wang et al. published results from small single-centre RCTs based in China (*n* = 17 and *n* = 36, respectively) [83, 84]. Whilst there were no differences in the number of lacunar infarcts between groups, they found significant reductions in post-treatment white matter hyperintensity volumes compared to baseline in the RIC groups. These results did not correspond to an overall improvement in cognition as measured by mini-mental state examination (MMSE) or MoCA, but the latter study detected an increase in visuo-spatial and executive MoCA subsections. These two studies found opposing results with regard to pulsatility index (PI), a measure that corresponds to downstream vessel damage, and one study detected a reduction in triglycerides, low-density lipoprotein (LDL), cholesterol and plasma homocysteine, suggesting that some of the effects of RIC may be attributed to risk factor modification in cSVD [84].

Another RCT of 42 patients with vascular dementia and Fazekas scores of ≥ 2 found a trend towards reduction in WMH volume and inflammatory markers (CRP, IL-6 and tumour necrosis factor-alpha; TNFα) in the RIC group at 6 months follow-up [85]. The RIC group had a greater improvement in visuospatial ability compared to the control group at 6 months (Judgment of Line Orientation cognitive testing). This study had the lowest-scoring baseline cognitive test measurements.

A study examining WMHs in patients with acute ischaemic stroke found a significant decrease in Fazekas scores at days 180 and 300 compared to baseline, as well as a reduction in Scheltens’ score at day 300 in RIC but not in control [86]. These radiological measures showed significant correlation to the improving cognitive scores (MMSE and MoCA) at all timepoints.

All of the studies are limited by their relatively small sizes, with the largest having 58 participants [86] and being single-centre based. Two studies were limited by unclear randomisation procedures [84, 85], and two raised the possibility of conflict of interest of the auto-control device being invented by a co-author [83, 84].

There are several possible reasons for the varying levels of correlation between radiological and cognitive improvement measures within the studies. There were differences in the driving disease processes across the study populations, their baseline cognition levels and SVD burden, and in the chosen modality for measuring radiological improvement (WMH volume vs Fazekas) and cognitive improvement. Further research is required to elucidate the mechanisms by which RIC affects cSVD progression, as well as determining whether any improvements are long-lasting.

### Carotid artery stenosis

The application of RIC prior to ischaemia is the original paradigm of ischaemic preconditioning [2]. In most circumstances, the occurrence of ischaemia cannot be predicted. However, ischaemic stroke is a recognised complication of carotid interventions including endarterectomy and stenting [87–89]. As such, RIPreC applied prior to carotid intervention has been investigated as a potential way to minimise the impact of stroke in this setting. Four studies evaluate the effect of RIC in carotid artery stenosis, and findings are summarised in Table 5.

**Table 5:** Comparison of studies of RIC in carotid artery stenosis

**Author**	**Population**	**RIC Protocol**	**Anaesthesia**	**New DWI lesions**	**Saccadic latency**	**Blood biomarkers**
Walsh	Elective CEA UK	Single cycle, 10 min BL thighs, Incremental pressure until doppler arterial signal lost	Propofol, midazolam, remifentanyl		↓	
Garcia	Elective CEA, AAA repair or PVD surgery US	3 cycles 5 min forearm, 12–24 h prior to surgery	Not reported			↔
Zhao	Elective CAS China	BL upper limb 5 cycles, 5 min, 200 mmHg, twice daily for 2 weeks prior to stenting	NA	↓^a^		↓
Asadi	Elective CAS Iran (37:37)	Single arm, 3 cycles, 5 min, 200 mmHg	NA	↓		

*DWI* Diffusion weighted imaging, *CEA* Carotid endarterectomy, *AAA* Abdominal aortic aneurysm, *PVD* Peripheral Vascular Disease, *CAS* Carotid artery stenting, *NA* not applicable

^a^reduction in lesion number and volume

Walsh et al. randomised 70 patients to sequential unilateral lower limb RIC in each leg for 10 min prior to undergoing carotid endarterectomy [90]. Given the occurrence of strokes post-carotid endarterectomy is uncommon, the authors used saccadic latency measured by quantitative oculometry as a surrogate marker of subtle cerebral injury. Whilst no TIA or strokes occurred in this study, a lower proportion of individuals in the RIC group had a deterioration in saccadic latency (32% vs 56%), although this was not statistically significant (*p* = 0.11). The CRIPES study looked at the safety and efficacy of RIC across a range of vascular interventions and, although they investigated RIC pre-carotid endarterectomy, there were no neurological or neuroimaging endpoints to be able to draw any conclusions on efficacy [91]. Zhao et al*.* reported that twice daily RIC for 2 weeks prior to carotid stenting was associated with a lower total diffusion-weighted imaging (DWI) lesion volume compared to sham RIC or standard medical therapy [92]. Asadi et al. demonstrated that a single instance of unilateral 3 × 5 min cycles at 200 mmHg of RIC 30 min before surgery causes a reduction in the number of patients with restricted lesions in DWI MRI, 15 (40.5%) in the intervention group and 19 (54.1%) in the control group; however, this reduction was not statistically significant [93]. The opposing results of Zhao et al. and Asadi et al. could be due to the different parameters of RIC, the effect of opting for bilateral over unilateral compression and single over multiple instances of RIC have not been explored thoroughly. These results suggest repeated cycles of bilateral compression cause a more potent neuroprotective response.

Given the possibility of clinically silent infarctions after carotid interventions [94], it will be useful to have further studies with DWI MRI as an outcome measure. Furthermore, more evidence is needed to determine whether several cycles of RIC in the days to weeks prior to carotid intervention is more beneficial than a single session of RIC immediately pre-surgery; hence, a comparison of single dose vs repeated dose RIC pre-carotid interventions would be welcomed.

### Aneurysmal subarachnoid haemorrhage

It is estimated that 15% of strokes are haemorrhagic in nature and result in high levels of morbidity [95]. Aneurysmal subarachnoid haemorrhage (aSAH) accounts for 5% of total strokes [95], and whilst major neurological damage occurs at the point of haemorrhage, there is the risk of delayed cerebral ischaemia (DCI) causing secondary damage from days 3 to 14 post-bleed [96, 97]. Several studies have focused on safety and feasibility of RIC in aSAH after surgical intervention and have examined the effects on DCI.

Phase I trials have confirmed the safety and tolerability of RIC, including no negative effects on blood coagulation or cerebral blood flow and no delayed ischaemic neurological deficits within 3 days of RIC [98, 99]. A 2011 RCT of 33 patients compared upper or lower limb RIC on a dose-escalation regime versus sham RIC [100]. RIC was found to be safe and well-tolerated as judged by subjective and objective measures. Three deep vein thromboses were reported in the RIC group, and although this was considered to be unrelated to the procedure, no clarification was given. The randomisation procedures were unclear and the practicalities of delivering RIC with intravenous lines present meant there was methodological variation with the site of cuff application.

A further study used alternating days of high-pressure RIC (20 mmHg above SBP) or low-pressure RIC (20 mmHg below SBP) for up to 6 sessions on days 2–12 post-SAH and compared this to retrospectively matched controls (*n* = 11 per group) [101]. The low-pressure group had shorter hospital and intensive care length of stay compared to control, but there was no difference in the primary outcome of vasospasm (measured by transcranial doppler), nor in mRS or mortality between any groups. Although distal pulses were present in the low-pressure group, the inflation could be sufficient to cause venous stasis, and the fact that the non-RIC controls were retrospectively matched means that this is difficult to investigate.

A 2015 study recruited 21 aSAH patients to undergo 4 sessions of RIC on alternate days and compared results to retrospectively matched controls [102]. They found that a higher proportion of those treated with RIC had favourable neurological outcomes at discharge, defined as mRS 0–2 (OR 5.17, 95% CI 1.21–25.02). To further investigate the humoral mechanisms potentially implicated in RIC, the same group examined DNA methylation and gene expression profiles pre- and post-RIC in 13 aSAH patients recruited as per the previous trial protocol [103]. They found changes in expression and methylation in several genes involved in the cell cycle and in inflammatory responses after RIC, although they comment that study limitations include that these changes may be attributed to other medical treatments, necessitating a non-RIC control group.

A 2021 RCT showed that RIPreC mitigates the development of cerebral vasospasm in aSAH patient [104]. All patients (*n* = 25) were investigated for cerebral vasospasm at baseline using transcranial Doppler and cerebral angiography. Patients were only re-assessed if clinical symptoms of vasospasm were evident. Thirteen patients were reinvestigated during the trial period, 4 in the RIC arm of which only 1 had a confirmed diagnosis of cerebral vasospasm and 9 in the control arm of which 8 had confirmed vasospasm on imaging, this difference was statistically significant. Furthermore, the RIC patient demonstrated mild vasospasm, and among the eight patients in the control group, three patients had mild, three had moderate, and two had severe angiographic vasospasm, further supporting the neuroprotective effect of RIC. This trial also explored the effect of RIC on S100B and neuron-specific enolase (NSE); previous studies have shown inconsistent results with regard to the prognostic and predictive potential of these biomarkers in aSAH patients; however, they have been shown to be reliable indicators of ischaemic damage [105–107]. Whilst there was no significant impact of RIC on biomarker levels, the variability seen in the data set suggests larger trials are needed to more definitively assess the effect of RIC on S100B and NSE. This study has also shown that RIC has a long-term neuroprotective effect, with patients showing significantly higher GOS scores at discharge and 6 months post-discharge; this further supports the positive effect on neurological outcomes of RIC as shown in previous studies. Whilst this trial has shown the potential of RIC therapy in aSAH, the small sample size calls for larger trials to validate these findings. Moreover, the unique RIC protocol used further highlights the need for additional experimentation to ascertain the optimal parameters of RIC therapy. Post hoc analysis of this trial revealed that RIC decreases cerebral oxygen desaturation (COD) in aSAH patients [108]. Regional cerebral oxygen saturation (rScO2) has been shown to be a reliable indicator of cerebral vasospasm [109]. In the RIPC group, the incidence of ipsilateral COD (a 20% reduction in rScO2 from baseline) was 15.4% (2 patients), compared to 33.3% (4 patients) in the sham group. There was a notable association between ipsilateral COD and poor GOS upon discharge. Whilst the precise mechanism underlying the improvement in rScO2 is not fully understood, it is thought to arise from enhanced pulmonary oxygenation, potentially linked to the upregulation of nitric oxide synthase and the release of endothelial nitric oxide attributed to RIC therapy [44].

### Intracerebral haemorrhage

Intracerebral haemorrhage (ICH) causes local tissue damage at the site of haematoma which can expand to cause mass effect and further rises in intracranial pressure. A secondary phase of injury is caused by perihaematomal oedema, which is associated with worse functional outcomes [110]. Early surgical clot evacuation is not recommended in supratentorial ICH; therefore, other treatment options are vital to explore [111, 112]. The use of RIC in intracerebral haemorrhage has not been widely studied but theoretical mechanisms of neuroprotection in animal models include reducing oedema through upregulation of factors that are protective against oedema [113].

RICH-1 is an open-label multi-centre randomised study of RIC in supratentorial ICH, comparing a once daily RIC protocol for 1 week to standard medical care (*n* = 20 in each group) [114]. Patients with ICH were included if GCS was above 8 and RIC was administered within 24–48 h of ICH onset. RIC was found to be safe, with no significant neurological deterioration by day 7 in either group, as measured by NIHSS and GCS. Whilst this was primarily an efficacy and safety trial, the authors also report imaging and functional outcomes. There was no significant difference in haematoma volume reduction at day 7 or in functional outcome at day 90, but the RIC group had a significantly higher haematoma resolution rate and relative perihaematomal oedema at day 7. A phase III RCT is underway (RICH-2, NCT04657133) that aims to detect favourable neurological outcomes as measured by mRS.

### Moyamoya disease

Moyamoya disease causes narrowing of the intracerebral arteries and subsequent development of fragile collateral circulation. It often presents in children and younger adults in the form of recurrent stroke [115]. It is treated surgically with anastomosis, which has risks of ischaemia in a similar manner to internal carotid artery surgery, as well as risks of hyper-perfusion syndrome leading to haemorrhage [115, 116]. Neuroprotective strategies have been investigated to reduce the risk of intra- and post-operative ischaemia–reperfusion injuries.

A single-arm open-label feasibility study published in 2019 recruited 30 children and adults with a history of ischaemic Moyamoya disease who had not undergone surgical bypass [117]. Long-term bilateral upper limb RIC was delivered three times daily. No RIC-related adverse effects occurred, and there was a suggestion of symptom improvement at 0.5-, 1- and 2-year follow-up on self-reported scoring. One patient had a new infarct at follow-up but there was a significantly reduced frequency of stroke recurrence and TIAs post-RIC, compared to pre-RIC. On perfusion studies, 75% showed improvement on SPECT and 95% on PET. This study was limited by the lack of a control group and a high drop-out rate, with fewer than half of the participants completing the 2-year follow-up.

A 2019 RCT randomised 108 patients with Moyamoya disease undergoing middle cerebral artery-superficial temporal artery anastomosis (MCA-STA) into 4 cycles of RIC (pre- and post-surgery) or into a sham RIC group [118]. The RIC-treated group had a shorter hospital length of stay. Major neurological complications (including stroke, SAH, seizures, and hyperperfusion) were lower in the RIC group vs control (5.6% vs 24%), with infarcts representing the majority of complications; 46.3% had TIA in the RIC group versus 51.9% in the control group, but this outcome was not included in the calculation for overall neurological complications.

A 2022 randomised control trial conducted by Xu et al. recruited 34 adult patients with Moyamoya disease [119]. The intervention group received bilateral upper limb RIC twice a day for 1 year, whilst the control group received standard medical and surgical treatment. This trial has shown significant improvement in the intervention group’s mean cBF compared to the control at 1 year; the control group showed a reduction in mean cBF of 0.03 ml/100 g per minute at 1 year when compared to baseline whilst the intervention group showed a significant improvement of 0.16 ml/100 g per minute. This supports the results of the single-arm study (2019) that RIC promotes cerebral perfusion. Whilst Xu et al. showed there was no difference in the features of periventricular anastomosis between both groups, the trial did show a significant reduction in arterial lesion progression; this is speculated to be due to the promotion of angiogenesis and arteriogenesis secondary to RIC.

Due to the low incidence of Moyamoya disease and the relative lack of research, there have been no major advancements in the management of the condition over the past decade [120]. These three trials provide valuable evidence to the potential therapeutic effect of RIC and provide the foundation for larger trials to verify the efficacy of RIC in the management of Moyamoya disease.

### Brain tumours

RIC has been studied as a neuroprotective method against post-operative ischaemia following elective brain tumour resection [121]. Sixty adults with primary or secondary brain tumours underwent 3 cycles of unilateral upper limb RIC or sham RIC after induction of anaesthesia. The incidence of new ischaemic lesions on 72-h post-op MRI was significantly lower in the RIC group compared to sham (63% vs 87%), but there was no difference in secondary outcome measures of mean infarct volume or in the incidence of postoperative neurological deficits. The authors reported a limitation in that the outcome analysis from primary and secondary brain tumours was not done separately. These conditions often have different rates of ischaemic complications due to their infiltrative properties requiring different surgical techniques. Additionally, this study was registered retrospectively, opening the door to reporting bias. Larger studies are required to determine how the ischaemic lesion process correlates with functional outcomes.

### Cervical spine surgery

One randomised controlled trial examined the effects of RIC on ischemia–reperfusion injury in patients with cervical spondylosis undergoing decompression surgery (*n* = 40) [122]. Although this study was not powered to detect an improvement in neurological outcome, the biochemical markers of neuronal damage and recovery rates were favourable in the RIC group. RIC significantly reduced serum S100B release at 6-h and 1-day post-surgery, with NSE reductions until day 5 post-surgery. Higher recovery rates (using the Japanese Orthopaedic Association Score of upper limb, lower limb and bladder function in cervical myelopathy) were seen at up to 3 months post-surgery in the RIC group than control group, but these differences equalised by month 6. This study is limited by unclear allocation concealment procedures, and although serum biomarkers are suggestive of reduced damage perioperatively, it remains to be seen whether this translates to reduced clinical complications.

### Traumatic brain injury

One trial of 40 patients investigated the effects of RIC on secondary brain injury post-TBI [123]. Secondary brain injury is thought to be mediated by inflammatory cascades within hours from the initial trauma and is thus potentially modifiable [124]. Adults with blunt TBI and intracranial haemorrhage, whose Glasgow Coma Score (GCS) was ≤ 8 were consecutively enrolled to RIC and standard medical care groups. Primary outcome measures were the levels of NSE and S-100B at 0-, 6- and 24-h post-RIC, and the study also looked at procedure safety. There were significant reductions of mean S-100B and NSE at 6 h and 24 h compared to baseline in the RIC group, whilst the control group showed increases in these biomarkers at the same time points. Paired comparisons showed statistically significant differences at 6- and 24-h time points between the groups. The procedure was not associated with local site complications, and there was no difference in neurosurgical intervention rates or mortality between the two groups. The authors postulated that secondary brain injury may be reduced through the anti-inflammatory effects of RIC, particularly concerning endothelial function and cerebral blood flow. However, the longer-term effects of RIC on these serum biomarkers and on neurological outcomes remains to be determined.

### Multiple sclerosis

There are a number of links between the ischaemic and inflammatory pathophysiological mechanisms of neurodegeneration in multiple sclerosis (MS) and the central effects of RIC [8]. Furthermore, peripheral effects of RIC on skeletal muscle vasculature may increase exercise tolerance [125, 126]. Only a single study has investigated RIC in MS; this demonstrated that a single cycle of RIC increased the distance walked in the 6MWT in individuals with MS. Given the effect of inflammatory demyelination on mobility and exercise tolerance, RIC is a potentially promising intervention to improve exercise capacity in individuals with MS [127]. The MPISC-2 trial (NCT03967106) is currently recruiting and investigating the effects of repeated cycles of self-delivered RIC on activity, fatigue and gait in MS. This will identify whether RIC can be self-delivered at home by MS patients and whether repeated cycles of RIC are more useful than a single cycle. Further studies are required to evaluate whether central effects of RIC on neuro-inflammation and cerebral blood flow affect the accumulation of disability in MS.

### Limitations

There are several limitations to the present study. First, there are methodological differences in the RIC protocols (principally cycle frequency, timing and duration) employed by studies within each condition which makes it difficult to directly compare results from separate trials. Second, the majority of studies have at least one factor which puts them at high risk of bias. Third, the majority of studies of stroke were performed in China where there is a higher risk of stroke from intracranial atherosclerosis compared to cardioembolic or carotid-embolic stroke; as such, the results from the detailed studies cannot necessarily be applied to other populations.

### Future directions

Over the past 5 years, there has been a remarkable increase in the number of RIC trials. Despite this surge in research, several uncertainties persist regarding treatment protocols and efficacy. The vast majority of work concentrates on conditions related to acute cerebrovascular disease; however, interest is now expanding to other neurological conditions including Parkinson's disease (NCT04327687), cerebral palsy (NCT04598711), and Moyamoya (NCT03546309).

A potential application of RIC lies in its use for the rehabilitation of chronic neurological conditions. Geng et al. compared the therapeutic effects of RIC and exercise as rehabilitation therapies after stroke in rats [128]. The results show RIC leads to significant improvement in infarct volume when compared to controls and exercise groups. Additionally, RIC outperforms or matches the improvements in neurological deficit, functional outcomes and cognitive deficit when compared to both mild and intense exercise groups. Furthermore, the study showed that the changes in protein expression seen in both RIC and exercise groups are very similar and therefore thought to induce their effects through common molecular pathways. Changes in molecular markers suggest both therapeutic options work by promoting neuroplasticity, synaptogenesis and angiogenesis [56, 128–132]. Exercise has been established as an effective rehabilitation method in stroke; studies have shown that it improves memory loss, neurological function and is neuroprotective [128–141]. However, compliance levels to exercise in post-stroke patients are low due to physical limitations, anxiety, lack of accessible facilities, lack of progress and the presence of comorbidities [142]. RIC provides a novel alternative that overcomes the limitations of exercise and provides similar beneficial effects and therefore could be a more impactful neurorehabilitation option. Several upcoming trials (NCT05263531, NCT03851302, NCT04598711, NCT05355883) will potentially provide human data to substantiate the efficacy of RIC in the rehabilitation of neurological conditions including stroke, cerebral palsy and spinal cord injury.

Current trial evidence indicates that a more intense RIC regimen results in more significant therapeutic impact [21]. However, there remains a scarcity of human trials exploring optimal RIC parameters, with the exception of a study by Kate et al. [40], which investigated the impact of 4 cycles compared to 6 cycles of RIC therapy. The results show no significant difference in infarct growth, neurological function or stroke recurrence between both groups. Nevertheless, further studies are needed to validate these findings. The existing animal studies suggest a dose–response correlation [143, 144], where chronic application of RIC appears to yield more significant effects compared to single application. However, analysis of current studies and additional trials are required to provide more conclusive evidence to support this correlation. TRIC-VCI (NCT04109963), an upcoming trial, will compare the effect of RIC administered once a day and twice a day for 30 days in patients with small vessel disease to determine its impact on cognitive impairment; the results will provide valuable information regarding optimal RIC parameters and will contribute to our understanding of the dose–response relationship of RIC. Presently, most trials utilise 5 cycles (24 trials), with earlier trials employing 4 cycles due to their adherence to the standard protocol utilised in animal studies and earlier cardiology trials. The recent shift to 5 cycles supports the dose–response correlation [132]. However, more studies are needed to identify the most appropriate number of cycle repetitions for optimal RIC outcomes. It has been observed that different mechanisms are activated during short-term RIC application, resulting in reduced infarct volume [145], whilst chronic RIC application induces enhanced cBF, angiogenesis and microvascular remodelling [49, 56, 132, 146, 147]. Further trials are needed to comprehensively understand the underlying mechanisms and to determine how they vary based on the timing of intervention and its duration; deepening our understanding of the mechanisms that underpin RIC will allow us to better utilise it in the management of neurological conditions and therefore maximise its therapeutic output.

As the evidence for RIC in neurology continues to expand and evolve, these upcoming trials hold considerable promise in elucidating the potential of RIC as a therapeutic intervention in various medical conditions. The insights gained from these studies could pave the way for more refined treatment protocols, improved patient outcomes and a deeper understanding of RIC's broader applications in neurology.

## Conclusions

There is evidence of a biological effect of remote ischaemic conditioning in the acute treatment of ischaemic stroke, the chronic treatment of symptomatic intracranial stenosis and many other neurological conditions. However, larger, adequately powered randomised controlled trials are required to prove efficacy on outcome measures of mortality, disability and recurrent stroke in these settings prior to adoption in clinical practice. There is also insufficient evidence of the effect of RIC in vascular cognitive impairment, subarachnoid haemorrhage, cervical spine surgery and pre-carotid endarterectomy and stenting to justify its use in clinical practice. Further, larger randomised controlled trials are required in a variety of clinical settings to establish the efficacy of RIC and the subgroups of patients who respond to RIC therapy and to identify the optimal dose, frequency and length of RIC therapy.

## Supplementary Information

Supplementary Material 1.

Supplementary Material 2.

Supplementary Material 3.

Supplementary Material 4.

## Data Availability

The datasets used and/or analysed during the current study are available from the corresponding author on reasonable request.

## Abbreviations

RIC: Remote ischaemic conditioning
RIPreC: Remote ischaemic preconditioning
RIPerC: Remote ischaemic perconditioning
RIPostC: Remote ischaemic postconditioning
RCT: Randomised controlled trial
mRS: Modified Rankin Scale
NIHSS: National Institute of Health Stroke Scale
cBF: Cerebral blood flow
CRP: C-reactive protein
SBP: Systolic blood pressure
ADL: Activity of daily living
TIA: Transient ischaemic attack
WMH: White matter hyperintensities
BDNF: Brain-derived neurotrophic factor
HSP-27: Heat shock protein 27
VEGF: Vascular endothelial growth factor
OTT: Onset to treatment time
FBG: Fasting blood glucose
GOS: Glasgow Outcome Score
GCS: Glasgow Coma Score
CNS: Central nervous system
MRI: Magnetic resonance imaging
MOCA: Montreal Cognitive Assessment
PHQ-9: Physical Health Questionnaire-9
PAI-1: Plasminogen activator inhibitor-1
FMD: Flow-mediated dilatation
PSCI: Post-stroke cognitive impairment
ICAM-1: Intracellular adhesion molecule-1
ET-1: Endothelin-1
SAP: Stroke-associated pneumonia
HLA-DR: Human leukocyte antigen-DR isotype
TLR: Toll-like receptor
IL-1B: Interleukin 1-Beta
IL-6: Interleukin-6
PSF: Post-stroke fatigue
FSS-7: Fatigue Severity Scale
6MWT: 6-Minute walk test
CPET: Cardiopulmonary exercise testing
P-MRS: Phosphorous-31 magnetic resonance spectroscopy
VE/VCO2: Minute ventilation/carbon dioxide slope
ATP: Adenosine triphosphate
cSVD: Cerebral small vessel disease
DSM-4: Diagnostic and Statistical Manual of Mental Disorders, 4th Edition
MMSE: Mini-Mental State Examination
PI: Pulsatility index
LDL: Low-density lipoprotein
TNFα: Tumour necrosis factor-alpha
AIS: Acute ischaemic stroke
MCI: Mild cognitive impairment
VLT: Verbal Learning Test
JLO: Judgment of Line Orientation Test
DWI: Diffusion-weighted imaging
aSAH: Aneurysmal subarachnoid haemorrhage
SAH: Subarachnoid haemorrhage
DCI: Delayed cerebral ischaemia
DNA: Deoxyribonucleic acid
NSE: Neuron-specific enolase
COD: Cerebral oxygen desaturation
rScO2: Regional cerebral oxygen saturation
ICH: Intracerebral haemorrhage
MCA-STA: Middle cerebral artery-superficial temporal artery anastomosis
MS: Multiple sclerosis
