# Supplementary figures and images for: Remote ischaemic conditioning for neurological disorders—a systematic review and narrative synthesis
Source: Syst Rev. 2024 Dec 19;13:308. doi: 10.1186/s13643-024-02725-8 (PMC11657452; doi:10.1186/s13643-024-02725-8)

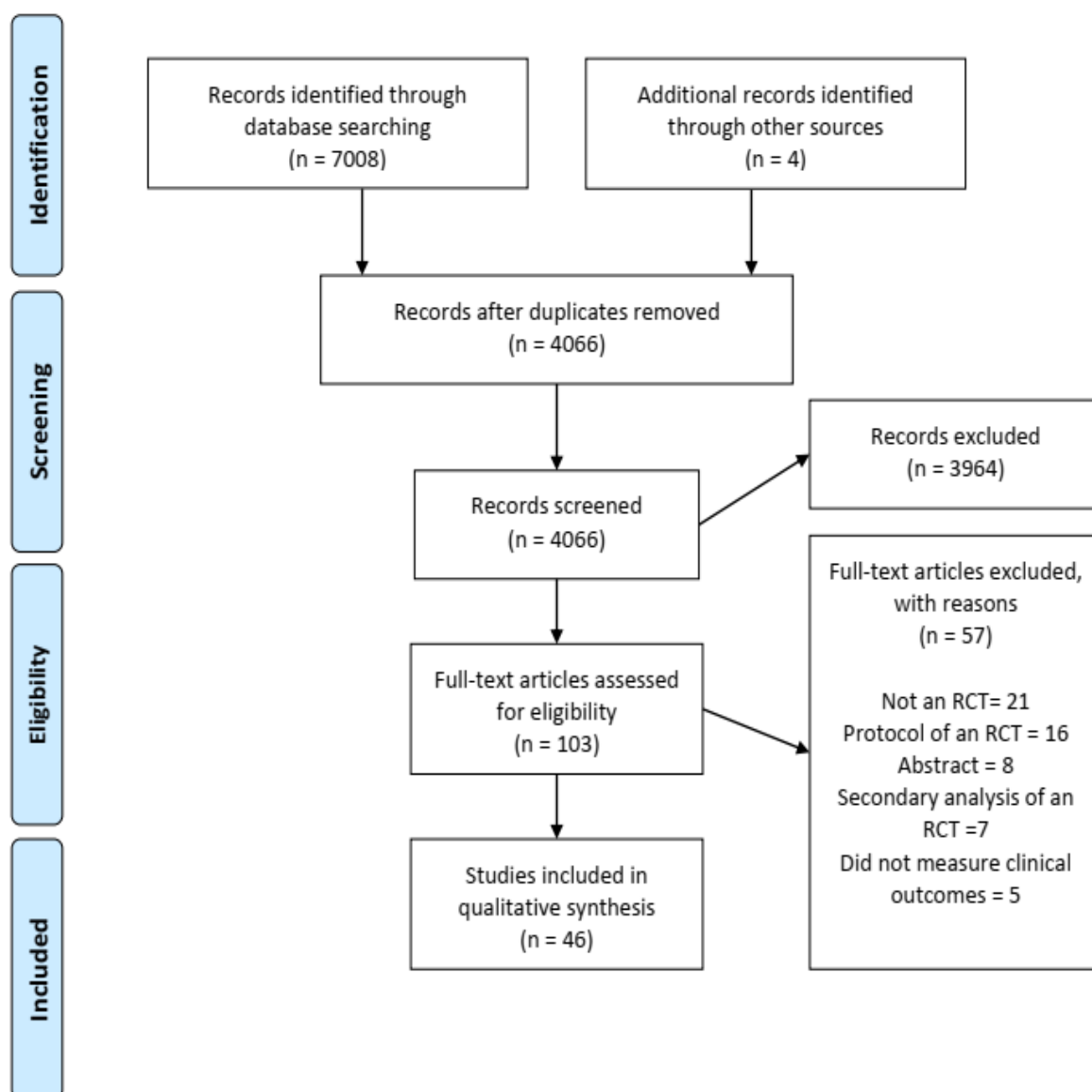

**Additional File 2.** Study selection flow diagram

Supplement: Supplementary file 2 — Supplementary Material 2. [file 13643_2024_2725_MOESM2_ESM.pdf]
