# Supplementary material for: Remote ischaemic conditioning for neurological disorders—a systematic review and narrative synthesis
Source: Syst Rev. 2024 Dec 19;13:308. doi: 10.1186/s13643-024-02725-8 (PMC11657452; doi:10.1186/s13643-024-02725-8)
Supplement: Supplementary file 3 — Supplementary Material 3. [file 13643_2024_2725_MOESM3_ESM.pdf]

Risk of bias

Study	D1	D2	D3	D4	D5	D6	Overall
Meng et al (2012)	?	?	X	+	X	X	X
Hougaard et al (2014)	+	+	X	+	X	X	X
Meng et al (2015)	+	+	+	+	X	X	X
Wei et al (2016)	?	?	X	+	+	+	X
England et al (2017)	+	+	X	+	+	+	X
Hyngstrom et al (2018)	?	?	X	X	+	+	X
Li et al (2018)	+	+	X	+	+	+	X
Xu et al (2018)	+	?	X	?	?	+	X
Che et al (2019)	+	+	X	+	+	X	X
Kate et al (2019)	+	+	X	+	X	X	X
England et al (2019)	+	+	X	+	+	+	X
Durand et al (2019)	+	+	X	X	+	X	X
Feng et al (2019)	+	?	X	?	X	+	X
Ji et al (2020)	?	?	X	?	?	+	X
Wei et al (2020)	+	+	?	X	X	X	X
An et al (2020)	X	X	X	+	+	+	X
Hyngstrom et al (2020)	+	+	+	+	+	+	+
Pico et al (2020)	+	+	X	+	+	+	X
Chen et al (2022)	+	X	X	+	+	+	X
He et al (2022)	+	X	X	?	+	+	X
Hou et al (2022)	+	+	+	+	+	+	+
Landman et al (2023)	+	+	X	+	X	+	X
Li et al (2020)	+	+	+	+	+	+	+
Poalelungi et al (2021)	+	+	+	+	+	+	+
Wang et al (2022)	+	+	X	+	+	+	X
Zhang et al (2021)	+	+	+	+	+	+	+
Moyle et al (2023)	+	+	X	+	+	+	X
Choi et al (2019)	+	+	X	+	+	+	X
Xu et al (2022)	+	+	X	+	+	+	+
Koch et al (2011)	?	?	?	?	+	?	?
Raval et al (2020)	+	+	+	+	+	+	+
Sangeetha et al (2021)	+	+	+	+	+	+	+
Walsh et al (2010)	+	+	X	X	X	+	X
Garcia et al (2016)	+	+	X	+	+	X	X
Zhao et al (2017)	?	?	X	+	X	X	X
Asadi et al (2022)	+	?	+	+	+	+	+
Healy et al (2015)	+	+	X	X	+	+	X
Joseph et al (2014)	X	X	?	?	+	?	X
Hu et al 2010	+	?	+	+	+	+	+
Mi et al (2016)	+	+	+	+	+	+	+
Zhou et al (2019)	+	+	X	+	X	X	X
Wang et al (2017)	?	+	+	+	X	X	X
Liao et al (2019)	?	?	+	+	+	X	X
Sales et al (2017)	+	+	+	+	+	?	+
Zhao et al (2020)	+	+	X	+	+	+	X
Chotiyarnwong et al (2020)	+	?	+	+	+	X	X

D1: Random Sequence Generation
D2: Allocation Concealment
D3: Blinding of participants and personnel
D4: Blinding of outcome assessment
D5: Incomplete outcome data (attrition bias)
D6: Selective reporting (reporting bias)

Judgement
X High
+ Low
? No information

Additional File 3. Risk of bias within studies
