# Supplementary material for: Remote ischaemic conditioning for neurological disorders—a systematic review and narrative synthesis
Source: Syst Rev. 2024 Dec 19;13:308. doi: 10.1186/s13643-024-02725-8 (PMC11657452; doi:10.1186/s13643-024-02725-8)
Supplement: Supplementary file 4 — Supplementary Material 4. [file 13643_2024_2725_MOESM4_ESM.pdf]

MEDLINE	Search Term	Results
1	isch* conditioning.ti,ab.	761
2	isch* preconditioning.ti,ab.	6,423
3	isch* perconditioning.ti,ab.	87
4	isch* postconditioning.ti,ab.	965
5	Ischemic Preconditioning.mp.	10,359
6	Ischemic Postconditioning.mp.	1,541
7	1 or 2 or 3 or 4 or 5 or 6	12,065
8	randomized controlled trial.pt.	592,915
9	controlled clinical trial.pt.	95,302
10	randomized.ti,ab.	655,656
11	placebo.ti,ab.	244,667
12	drug therapy.fs.	2,590,832
13	randomly.ti,ab.	409,265
14	trial.ti,ab.	751,633
15	groups.ti,ab.	2,544,587
16	8 or 9 or 10 or 11 or 12 or 13 or 14 or 15	5,724,959
17	exp animals/ not humans.sh.	5,121,777
18	16 not 17	4,999,827
19	7 and 18	1,955

EMBASE	Search Term	Results
1	isch* conditioning.ti,ab.	1,128
2	isch* preconditioning.ti,ab.	8,417
3	isch* perconditioning.ti,ab.	130
4	isch* postconditioning.ti,ab.	1,409
5	ischemic preconditioning.sh.	7,956
6	1 or 2 or 3 or 4 or 5	13,711
7	randomised control trial.pt.	0
8	controlled clinical trial.pt.	0
9	randomized.pt,ab.	895,366
10	placebo.ti,ab.	365,565
11	drug therapy.sh.	977,169
12	randomly.ti,ab.	549,794
13	trial.ti,ab.	1,115,247
14	groups.ti,ab.	3,587,921
15	7 or 8 or 9 or 10 or 11 or 12 or 13 or 14	5,727,594
16	(animals not humans).sh.	396
17	15 not 16	5,727,547
18	6 and 17	5,047

Additional File 4. Search strategy for use in OVID MEDLINE and EMBASE
